# Circulating Thyroxine, Thyroid-Stimulating Hormone, and Hypothyroid Status and the Risk of Prostate Cancer

**DOI:** 10.1371/journal.pone.0047730

**Published:** 2012-10-30

**Authors:** Alison M. Mondul, Stephanie J. Weinstein, Tracey Bosworth, Alan T. Remaley, Jarmo Virtamo, Demetrius Albanes

**Affiliations:** 1 Nutritional Epidemiology Branch, Division of Cancer Epidemiology and Genetics, National Cancer Institute, National Institutes of Health (NIH), Department of Health and Human Services, Bethesda, Maryland, United States of America; 2 Laboratory Medicine Department, NIH Clinical Center, National Institutes of Health, Bethesda, Maryland, United States of America; 3 Department of Chronic Disease Prevention, National Institute for Health and Welfare, Helsinki, Finland; National University of Ireland Galway, Ireland

## Abstract

**Background:**

Thyroid hormones may influence risk of cancer through their role in cell differentiation, growth, and metabolism. One study of circulating thyroid hormones supports this hypothesis with respect to prostate cancer. We undertook a prospective analysis of thyroid hormones and prostate cancer risk in the Alpha-Tocopherol, Beta-Carotene Cancer Prevention (ATBC) Study.

**Methods:**

Within the ATBC Study, a randomized controlled trial of α-tocopherol and β-carotene supplements and cancer incidence in male smokers, 402 prostate cancer cases were sampled. Controls were matched 2∶1 to cases on age and date of blood collection. Odds ratios (OR) and 95% confidence intervals (CI) of prostate cancer were estimated for quintiles of serum total and free thyroxine (T4), thyroid-stimulating hormone (TSH), thyroid-binding globulin (TBG), and by categories of thyroid status.

**Results:**

Men with serum higher TSH had a decreased risk of prostate cancer compared to men with lower TSH (Q5 vs. Q1–4: OR = 0.70, 95% CI: 0.51–0.97, *p = 0.03*). When the T4 and TSH measurements were combined to define men as hypothyroid, euthyroid or hyperthyroid, hypothyroid men had a lower risk of prostate cancer compared to euthyroid men (OR = 0.48, 95% CI = 0.28–0.81, *p = 0.006*). We observed no association between hyperthyroid status and risk, although the number of hyperthyroid men with prostate cancer was small (n = 9).

**Conclusions:**

In this prospective study of smokers, men with elevated TSH and those classified as being in a hypothyroid state were at decreased risk of prostate cancer. Future studies should examine the association in other populations, particularly non-smokers and other racial/ethnic groups.

## Introduction

**Figure 1:**
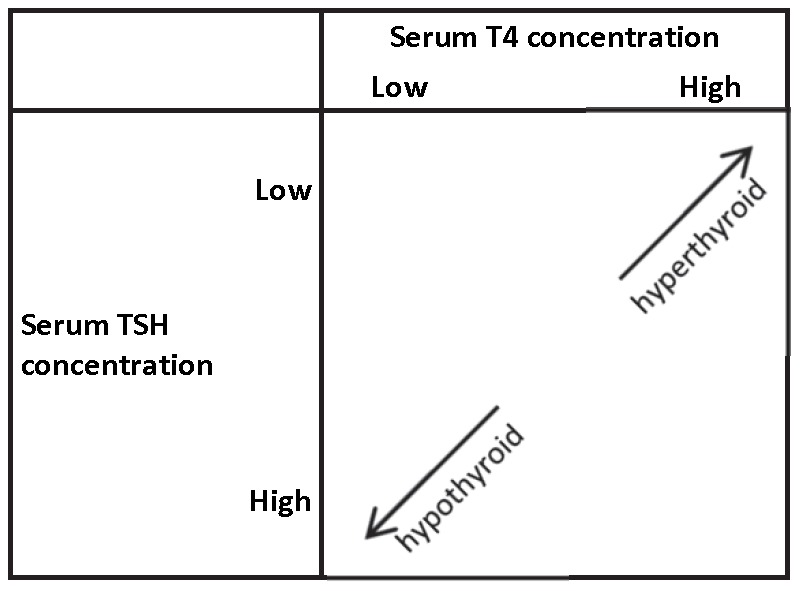
Hypothyroid and hyperthyroid hormone profiles by serum concentrations of T4 and TSH.

The thyroid hormones triiodothyronine (T3) and its prohormone thyroxine (T4) are hypothesized to promote carcinogenesis through their important role in cell differentiation, growth, and metabolism [1]. The hormones also promote tumor induced angiogenesis [2], and have been shown to increase prostate cancer cell proliferation *in vitro* [3], [4]. Thyroid-stimulating hormone (TSH) is produced by the anterior pituitary gland in order to regulate T4 secretion from the thyroid and is an important laboratory measure for determining thyroid status [5]. In individuals with normal thyroid function, T4 and TSH act in a negative feedback loop [5]; thus, a hypothyroid state is defined as having low T4 but high TSH, and hyperthyroid status is defined as having high T4 but low TSH (Figure 1) [6]. It is hypothesized that men who are hypothyroid may be at a decreased risk of prostate cancer, whereas hyperthyroid men may have an increased risk.

Few clinical or epidemiologic studies have examined this hypothesis. One cross-sectional study found that compared to men with low-grade prostate cancer or benign prostatic hyperplasia (BPH), those with Gleason ≥8 disease had elevated TSH levels [7]. Similarly, another cross-sectional study showed higher circulating T3 in prostate cancer cases compared to controls [8]. Only two prospective studies examined the relation between thyroid hormones or status and risk of prostate cancer [9], [10]. One observed that men with self-reported thyroid disease were at increased risk of prostate cancer, but this study did not differentiate between hypothyroid or hyperthyroid states [9]. The only study of circulating thyroid hormone levels and prostate cancer found an inverse association between TSH concentration and risk (advanced cases were not examined separately) [10].

We therefore conducted a prospective analysis in the Alpha-Tocopherol, Beta-Carotene Cancer Prevention (ATBC) Study to examine the association between individual circulating thyroid hormones, as well as thyroid status, and risk of prostate cancer.

## Materials and Methods

### Study Population

The ATBC Study (clincailtrials.gov identifier: NCT00342992) was a randomized, double-blind, placebo-controlled, primary prevention trial conducted to examine the effects of supplementation with α-tocopherol and β-carotene on cancer incidence [11]. Caucasian male smokers (n = 29,133) from southwestern Finland were recruited between 1985 and 1988. Men were between 50–69 years old at baseline and smoked at least 5 cigarettes per day as part of the enrollment criteria. Men were ineligible if they had previously had cancer or another serious illness at enrollment, or if they reported current use of supplements containing vitamin E (>20 mg), vitamin A (>20,000 IU), or β-carotene (>6 mg). Men who were enrolled in the trial were assigned to one of four groups based on a 2×2 factorial design: 1) α-tocopherol (dl-α-tocopheryl acetate, 50 mg/day), 2) β-carotene (20 mg/day), 3) both supplements, or 4) placebo. Trial participants were supplemented for 5–8 years, until death, or until the trial ended on April 30, 1993. Although the trial has ended, follow-up is ongoing through the Finnish Cancer Registry and the Register of Causes of Death. At enrollment, participants completed questionnaires about general risk factors, smoking, and medical history, as well as a food-frequency questionnaire. Participants also had height and weight measured and an overnight fasting blood sample collected.

Prostate cancer cases were identified by linkage with the Finnish Cancer Registry, which provides nearly 100% complete incident cancer ascertainment in Finland [12]. Medical records for the cases diagnosed prior to September 2001 were reviewed by one or two study oncologists to confirm diagnosis and staging, with subsequent cases through April, 2005 based only on Finnish Cancer Registry data. We randomly sampled 402 prostate cancer cases from those that were diagnosed at least 3 years after baseline, in order to minimize the possibility of reverse causation. Controls were sampled from ATBC Study participants who were alive and cancer-free at the time the case was diagnosed and were matched 2∶1 with cases on age (+/−1 year) and date of blood collection (±30 days). After excluding 3 cases and 2 controls with insufficient sample for analysis, 401 case-control sets (n = 800 controls) remained for analysis. Cases were defined as “aggressive” (n = 76) if they were TNM stage III or IV, AJCC stage 3 or higher, or Gleason sum 8 or higher. Stage or Gleason sum information was available for 57% of the cases.

### Exposure Assessment

Fasting serum samples were collected at baseline and were stored at −70°C. TSH, T4, and thyroid-binding globulin (TBG), the major carrier of T4 in circulation, were measured using the Immulite 2500 immunoassay system in the laboratory of Dr. Alan Remaley in the NIH Clinical Center. Each batch of samples contained four to six blinded quality control (QC) samples of pooled serum from the ATBC Study. The inter- and intra-batch CVs were as follows: 6.6% and 8.0%, respectively for T4; 3.3% and 5.2%, respectively for TSH; 5.1% and 8.8%, respectively for TBG. The assay reference ranges for T4, TSH, and TBG, respectively, were as follows: 4.0–12.5 µg/dL, 0.4–4.0 µIU/mL, 13–39 µg/mL. Free T4 was calculated from measured total T4 and TBG using a mass action equation [13].

### Statistical Analysis

Conditional logistic regression was used to estimate odds ratios and 95% confidence intervals of prostate cancer by quintiles of total T4, free T4, TSH, and TBG. We evaluated the trend across categories by modeling the median of each category as a continuous variable and evaluating its statistical significance using the Wald test. We also examined the association between hypo- or hyperthyroidism vs. normal thyroid function and risk of prostate cancer. There were too few men with clinical hypo- or hyperthyroidism to examine clinical and sub-clinical disease separately. Therefore, we grouped clinical or sub-clinical disease together, and defined a hypothyroid state as high TSH levels and normal or low T4 levels, and hyperthyroid status as low TSH with normal or high T4 levels. The normal range for TSH was defined as 0.3–3 µIU/mL and the normal range for T4 was defined as 4.6–12 µg/dL. We chose to define the normal range for TSH more narrowly than the assay reference range based on an American Association of Clinical Endocrinologists recommendation favoring this range for defining normal thyroid function [6]. All models were conditioned on the matching factors (age and date of blood collection).

The following factors that are hypothesized or known to be associated with either prostate cancer or thyroid hormone levels were included in the multivariable models: serum concentrations of total cholesterol, α-tocopherol, β-carotene, and retinol, cigarettes per day, years smoked, family history of prostate cancer, leisure time physical activity, body mass index (BMI), height, education level, marital status, urban residence, and intake of total energy, fruit, vegetables, red meat, alcohol, dietary vitamin D, and supplemental calcium. Further adjustment for the randomized trial intervention assignment (α-tocopherol or β-carotene treatment group) did not alter the results. PSA measurements are not included because PSA screening was not available at the time this cohort was established, and continues to be uncommon in Finland.

For thyroid hormones showing or suggesting a main effect association, we conducted stratified analyses by categories (<median vs. ≥median, except intervention status and family history) of age, α-tocopherol and β-carotene intervention assignment, height, BMI, cigarettes per day, baseline and follow-up serum retinol, family history of prostate cancer, and time between blood draw and case diagnosis. Stratified analyses were conducted using unconditional logistic regression adjusting for the matching factors. The main model results were unchanged when this approach was used instead of conditional logistic regression, making biased estimates unlikely. Statistical interaction was assessed using the likelihood ratio test.

### Ethics Statement

The ATBC study was approved by institutional review boards at both the US National Cancer Institute and the Finnish National Public Health Institute, and written informed consent was obtained from all participants.

## Results

Characteristics of the study population by case status are shown in Table 1. Cases and controls did not differ markedly with the exception of cases being more likely to have had a family history of prostate cancer and to consume fewer fruits and vegetables (Table 1). Characteristics of the study population by quintiles of serum T4 and serum TSH are shown in Tables 2 and 3, respectively. Men with higher T4 tended to be older, have higher serum cholesterol, α-tocopherol, and β-carotene and lower serum retinol, were more educated and physically active, more likely to be married or use dietary supplements and less likely to live in an urban environment, and had lower alcohol consumption than men with lower T4 (Table 2). Men with higher TSH tended to have higher serum cholesterol, α-tocopherol, and retinol, lower β-carotene concentrations, were less physically active, more educated and likely to live in an urban environment, had lower red meat consumption but higher alcohol consumption, and more likely to take dietary supplements than men with lower TSH (Table 3).

**Table 1:** Age-adjusted* baseline† characteristics by case-control status.

	Controls	Cases	*p-value*
**N**	401	800	
**Age (years)**	57.3	57.3	matched
**Height (cm)**	174	174	*0.72*
**Weight (kg)**	79.1	79.2	*0.72*
**BMI (kg/m^2^)**	26.2	26.2	*0.61*
**Serum cholesterol (mmol/L)**	6.2	6.3	*0.34*
**Serum α-tocopherol (mg/L)**	12.0	11.9	*0.63*
**Serum β-carotene (µg/L)**	221	225	*0.42*
**Serum retinol (µg/L)**	591	601	*0.21*
**Cigarettes per day**	19.5	19.8	*0.32*
**Years of smoking**	35.0	35.8	*0.13*
**Family history of prostate cancer**			
(%)	3.3	5.2	*0.08*
**Physically active**			
(%)	22.1	21.5	*0.79*
**>Elementary school education**			
(%)	25.8	26.4	*0.80*
**Married**			
(%)	79.8	79.1	*0.78*
**Urban residence**			
(%)	61.8	60.1	*0.58*
**Dietary intake per day**			
**Total energy (kcal)**	2,712	2,691	*0.92*
**Total fat (g)**	101	103	*0.19*
**Vitamin A (µg)**	1,483	1,517	*0.27*
**Vitamin D (µg)**	5.3	5.1	*0.11*
**Calcium (mg)**	1,391	1,422	*0.23*
**Fruit (g)**	223	201	*0.05*
**Vegetables (g)**	302	285	*0.02*
**Red meat (g)**	70.7	68.5	*0.44*
**Alcohol (ethanol, g)**	16.7	15.7	*0.26*
**Supplement use**			
**Vitamin A**			
**%**	12.3	12.1	*0.89*
**Vitamin D**			
**%**	7.4	7.0	*0.80*
**Calcium**			
**%**	11.3	12.3	*0.62*

* - Values are means unless otherwise indicated.

† - All characteristics are from the baseline questionnaire except family history which was collected during follow-up and is available for 889 men in this case control set. Baseline dietary data were available for 1,117 men, and 246 men claimed supplement use at baseline.

**Table 2:** Age-adjusted* baseline† characteristics by quintile of baseline serum thyroxine (T4).

	Quintile of serum thyroxine (T4) (µg/dL)				
	Q1<6.5	Q2 6.5 - <7.4	Q3 7.4 - <8.3	Q4 8.3 - <9.2	Q5≥9.2
**Age**	56.4	56.8	57.4	57.6	58.4
**Height (cm)**	174	174	173	174	174
**Weight (kg)**	79.8	78.9	79.2	79.5	78.8
**BMI (kg/m^2^)**	26.5	26.1	26.3	26.0	26.0
**Serum cholesterol (mmol/L)**	6.0	6.3	6.3	6.3	6.4
**Serum α-tocopherol (mg/L)**	11.1	12.1	12.2	12.0	12.4
**Serum β-carotene (µg/L)**	214	219	218	225	236
**Serum retinol (µg/L)**	606	608	586	589	580
**Cigarettes per day**	19.4	20.1	20.4	18.9	19.3
**Years of smoking**	34.1	35.1	36.0	35.1	36.1
**Family history of prostate cancer**					
%	6.4	5.7	5.0	4.2	5.7
**Physically active**					
%	20.3	21.0	21.1	22.0	25.0
**>Elementary school education**					
%	21.3	25.5	27.3	28.8	26.2
**Married**					
%	73.3	82.4	80.9	81.9	80.2
**Urban residence**					
%	63.4	59.5	61.4	62.0	57.6
**Dietary intake per day**					
**Total energy (kcal)**	2,705	2,663	2,710	2,730	2,693
**Total fat (g)**	101	99	102	104	102
**Vitamin A (µg)**	1,441	1,538	1,509	1,566	1,413
**Vitamin D (µg)**	5.2	5.1	5.1	5.3	5.4
**Calcium (mg)**	1,419	1,338	1,434	1,428	1,398
**Fruit (g)**	220	206	212	219	223
**Vegetables (g)**	293	299	303	294	290
**Red meat (g)**	66.1	73.3	74.4	66.1	68.3
**Alcohol (ethanol, g)**	20.5	18.6	15.6	15.2	11.6
**Supplement use**					
**Vitamin A**					
**%**	6.1	15.6	14.2	12.1	13.6
**Vitamin D**					
**%**	4.1	9.2	7.1	8.7	7.3
**Calcium**					
**%**	7.9	15.1	13.8	10.8	10.8

* - Directly standardized to the age distribution of the entire cohort. Values are means unless otherwise indicated.

† - All characteristics are from the baseline questionnaire except family history which was collected during follow-up and is available for 889 men in this case control set. Baseline dietary data were available for 1,117 men, and 246 men claimed supplement use at baseline.

**Table 3:** Age-adjusted* baseline† characteristics by quintile of baseline serum thyroid stimulating hormone (TSH).

	Quintile of serum thyroid stimulating hormone (TSH) (µIU/mL)				
	Q1<0.8	Q2 0.8 - <1.1	Q3 1.1 - <1.5	Q4 1.5 - <2.2	Q5≥2.2
**Age**	58.0	57.5	57.1	56.7	57.4
**Height (cm)**	174	174	174	174	174
**Weight (kg)**	78.2	78.7	79.4	79.9	80.0
**BMI (kg/m^2^)**	25.8	26.1	26.1	26.5	26.4
**Serum cholesterol (mmol/L)**	6.1	6.2	6.4	6.3	6.3
**Serum α-tocopherol (mg/L)**	11.4	11.9	12.2	12.3	12.1
**Serum β-carotene (µg/L)**	237	231	236	206	203
**Serum retinol (µg/L)**	572	590	603	595	615
**Serum total T4**	7.8	7.8	7.8	8.2	7.7
**Serum TBG**	19.6	19.6	20.5	21.2	21.0
**Serum free T4**	1.7	1.7	1.6	1.7	1.5
**Cigarettes per day**	20.5	19.6	19.4	19.0	19.5
**Years of smoking**	35.6	35.3	35.5	35.2	34.8
**Family history of prostate cancer**					
%	5.5	6.0	4.3	5.3	5.3
**Physically active**					
%	23.8	20.4	23.1	24.5	17.5
**>Elementary school education**					
%	21.6	21.1	28.6	31.7	26.7
**Married**					
%	81.5	79.7	80.8	79.2	78.0
**Urban residence**					
%	51.5	64.5	63.4	65.2	61.3
**Dietary intake per day**					
**Total energy (kcal)**	2,718	2,673	2,720	2,710	2,710
**Total fat (g)**	104	99.1	103	101	102
**Vitamin A (µg)**	1,521	1,463	1,532	1,530	1,420
**Vitamin D (µg)**	5.0	5.5	4.9	5.6	5.2
**Calcium (mg)**	1,434	1,346	1,437	1,404	1,400
**Fruit (g)**	207	223	205	233	212
**Vegetables (g)**	293	306	294	305	282
**Red meat (g)**	71.0	71.6	68.0	72.6	65.5
**Alcohol (ethanol, g)**	13.0	16.3	16.8	15.2	20.7
**Supplement use**					
**Vitamin A**					
**%**	7.9	14.2	14.5	13.9	10.3
**Vitamin D**					
**%**	3.7	10.8	7.8	6.4	7.5
**Calcium**					
**%**	7.7	16.7	12.3	12.0	9.2

* - Directly standardized to the age distribution of the entire cohort. Values are means unless otherwise indicated.

† - All characteristics are from the baseline questionnaire except family history which was collected during follow-up and is available for 889 men in this case control set. Baseline dietary data were available for 1,117 men, and 246 men claimed supplement use at baseline.

We observed a weak positive association between total and free T4 and risk of overall prostate cancer that was not statistically significant (Table 4). The association for total T4 appeared stronger for aggressive cases, and for both overall and aggressive prostate cancer, the associations were attenuated somewhat with multivariable adjustment (Table 4). There was no association between TBG and risk of either overall or aggressive prostate cancer (Table 4).

**Table 4:** Association between thyroid hormones and risk of overall and aggressive prostate cancer.

	Overall Prostate Cancer			Aggressive* Prostate Cancer		
	# Cases/# Controls	OR (95% CI)†	OR (95% CI)‡	# Cases/# Controls	OR (95% CI)†	OR (95% CI)‡
**Thyroxine (T4) (µg/dL)**						
Q1: <6.5	75/163	1.0 (ref)	1.0 (ref)	9/163	1.0 (ref)	1.0 (ref)
Q2: 6.5 - <7.4	78/164	1.03 (0.71–1.52)	0.96 (0.65–1.44)	18/164	1.97 (0.86–4.52)	1.77 (0.74–4.26)
Q3: 7.4 - <8.3	80/160	1.09 (0.74–1.60)	1.11 (0.74–1.66)	14/160	1.56 (0.65–3.70)	1.53 (0.62–3.79)
Q4: 8.3 - <9.2	82/156	1.17 (0.79–1.74)	1.14 (0.75–1.74)	16/156	1.82 (0.78–4.25)	1.77 (0.73–4.28)
Q5: ≥9.2	86/157	1.22 (0.82–1.83)	1.15 (0.75–1.77)	19/157	2.10 (0.92–4.83)	1.85 (0.77–4.45)
*p-trend*		*0.27*	*0.39*		*0.15*	*0.25*
**Free thyroxine (Free T4) (ng/dL)**						
Q1: <1.4	70/170	1.0 (ref)	1.0 (ref)	14/170	1.0 (ref)	1.0 (ref)
Q2: 1.4 - <1.6	83/163	1.26 (0.86–1.85)	1.28 (0.85–1.92)	17/163	1.26 (0.60–2.65)	1.20 (0.56–2.58)
Q3: 1.6 - <1.7	66/138	1.18 (0.78–1.79)	1.21 (0.79–1.86)	13/138	1.14 (0.52–2.50)	1.10 (0.48–2.51)
Q4: 1.7 - –<1.9	99/170	1.46 (0.99–2.14)	1.55 (1.03–2.33)	18/170	1.27 (0.61–2.25)	1.04 (0.48–2.24)
Q5: ≥1.9	83/159	1.33 (0.88–2.01)	1.33 (0.86–2.07)	14/159	1.04 (0.48–2.25)	0.76 (0.33–1.74)
*p-trend*		*0.16*	*0.21*		*0.97*	*0.42*
**Thyroid-binding globulin (TBG) (µg/mL)**						
Q1: <16.1	76/155	1.0 (ref)	1.0 (ref)	13/155	1.0 (ref)	1.0 (ref)
Q2: 16.1 - <18.7	77/169	0.93 (0.64–1.37)	0.95 (0.64–1.42)	10/169	0.71 (0.30–1.67)	0.67 (0.27–1.64)
Q3: 18.7 - <21.3	83/152	1.12 (0.76–1.66)	1.08 (0.72–1.64)	17/152	1.31 (0.62–2.80)	1.54 (0.69–3.42)
Q4: 21.3 - <24.4	84/163	1.07 (0.71–1.59)	1.09 (0.71–1.67)	21/163	1.52 (0.73–3.14)	1.79 (0.82–3.89)
Q5: ≥24.4	81/161	1.03 (0.68–1.56)	1.04 (0.66–1.62)	15/161	1.08 (0.50–2.36)	1.18 (0.51–2.73)
*p-trend*		*0.72*	*0.73*		*0.40*	*0.26*
**Thyroid stimulating hormone (TSH) (µIU/mL)**						
Q1: <0.8	83/158	1.0 (ref)	1.0 (ref)	12/158	1.0 (ref)	1.0 (ref)
Q2: 0.8–<1.1	84/157	1.02 (0.70–1.49)	1.01 (0.68–1.50)	18/157	1.55 (0.72–3.33)	1.47 (0.66–3.29)
Q3: 1.1 - <1.5	84/167	0.96 (0.66–1.40)	0.95 (0.64–1.41)	18/167	1.43 (0.67–3.07)	1.37 (0.62–3.02)
Q4: 1.5 - <2.2	83/151	1.04 (0.71–1.52)	1.08 (0.73–1.62)	15/151	1.35 (0.61–2.98)	1.38 (0.59–3.19)
Q5: ≥2.2	67/167	0.77 (0.53–1.14)	0.71 (0.47–1.06)	13/167	1.03 (0.45–2.32)	0.93 (0.39–2.20)
*p-trend*		*0.17*	*0.10*		*0.67*	*0.54*
**Clinical thyroid status** §						
Hypothyroid	20/75	0.52 (0.31–0.85)	0.48 (0.28–0.81)	-	-	-
Euthyroid	372/704	1.0 (ref)	1.0 (ref)	-	-	-
Hyperthyroid	9/19	0.92 (0.41–2.05)	0.86 (0.37–1.99)	-	-	-

* - Information on cancer stage and grade available for cases diagnosed through July 2002.

† - Conditioned on age and date of baseline blood draw.

‡ - Conditioned on age and date of baseline blood draw. Further adjusted for body mass index (kg/m2), serum concentrations of retinol, total cholesterol, alpha-tocopherol, and beta-carotene, cigarettes smoked per day, years smoked, family history of prostate cancer, physical activity, education, marital status, urban residence, total intake of energy, dietary vitamin D, fruit, vegetables, red meat, alcohol, and use of calcium supplements.

§ - Hypothyroid defined as TSH >3 µIU/mL and T4 <4.6 µg/dL. Hyperthyroid defined as TSH <0.3 µIU/mL and T4 >12 µg/dL.

There was a suggestion that TSH was related to risk of prostate cancer overall, but not to aggressive disease (Table 4). Men in the highest quintile of TSH were at approximately 30% lower risk than men in the lowest quintile, while men in quintiles 2–4 appeared to have risk similar to that of men in the 1^st^ quintile (Table 4). Dividing the top quintile into two deciles defined by TSH ≥2.85 µIU/mL showed OR's and 95% CI's of 0.79 (0.45–1.37) and 0.52 (0.29–0.95) for deciles 9 and 10 (vs. decile 1), respectively. Combining quintiles 1–4 for a referent category showed that men with TSH ≥2.2 µIU/mL had a statistically significantly reduced risk of prostate cancer compared to men with TSH<2.2 µIU/mL (multivariable-adjusted OR = 0.70, 95% CI: 0.51–0.97, *p = 0.03*).

When the T4 and TSH measurements were combined to define men as being in a hypothyroid, euthyroid or hyperthyroid hormonal state, we found that men who were hypothyroid (i.e., TSH≥3.0 µIU/mL and T4<4.6 µg/dL) had a statistically significantly reduced risk of overall prostate cancer compared to euthyroid men (Table 4). We observed no association between hyperthyroid status and risk, although the number of cases in this group was small (Table 4). There were too few cases of aggressive prostate cancer in either the hypothyroid or hyperthyroid categories to examine them separately (i.e., n = 4 and n = 1, respectively). When we used the more stringent assay reference range to define men as hyper- or hypothyroid, we observed a similar reduced risk of prostate cancer for hypothyroid men (OR = 0.55, 95% CI = 0.28–1.08, *p = 0.08*).

Exploratory analyses of T4 that stratified by potential effect modifiers revealed a stronger, statistically significant positive association with prostate cancer risk among men who received the ATBC trial β-carotene supplement (Q5 vs. Q1 OR = 1.82, 95% CI = 1.03–3.24) as compared with those who were not supplemented (Q5 vs. Q1 OR = 0.68, 95% CI = 0.39–1.20, *p for interaction = 0.02*). Also, the positive association may have been limited to men who were taller, although the interaction was not statistically significant (*p for interaction = 0.17*; data not shown). Stratified analyses examining TSH showed that the inverse association was limited to men who were older (≥57 years: Q5 vs. Q1 OR = 0.53, 95% CI = 0.30–0.92; <57 years: Q5 vs. Q1 OR = 1.09, 95% CI = 0.60–1.98, *p for interaction = 0.002*). The inverse association between TSH and prostate cancer appeared stronger among men who smoked fewer cigarettes daily (<20 cigarettes: Q5 vs. Q1 OR = 0.60, 95% CI = 0.31–1.09; ≥20 cigarettes: Q5 vs. Q1 OR = 0.86, 95% CI = 0.52–1.45, *p for interaction = 0.08*). We also observed statistically significant inverse associations between TSH and risk among men who had lower serum retinol and those randomized to the trial β-carotene supplement, although these interactions were not statistically significant (*p for interaction = 0.13 and 0.27*, respectively). Too few cases were categorized as hyperthyroid to permit subgroup analyses. Because of the possible interactions with the trial β-carotene intervention for T4 and TSH, however, we examined the association between hypothyroid status and risk of prostate cancer stratified by β-carotene supplementation and found no difference (hypothyroid vs. euthyroid: no β-carotene OR = 0.49, 95% CI = 0.23–1.04; β-carotene supplemented OR = 0.46, 95% CI = 0.22–0.96; *p for interaction = 0.93*).

## Discussion

We observed that men with clinical or subclinical hypothyroid status as defined based on recent endocrinological guidelines [6] were at decreased risk of prostate cancer compared to men with normal thyroid function. Similarly, we observed that men with the highest concentrations of TSH (indicating a hypothyroid state) were at lower risk of prostate cancer. These findings are consistent with previous laboratory and epidemiologic data suggesting that thyroid hormones influence prostate cancer risk [3], [4], [7]–[10]. A well-characterized biological mechanism through which this may occur involves T4 and T3 binding to the plasma membrane receptor integrin αvβ3, which activates various pro-carcinogenic pathways, including PI-3K and MAPK/ERK1/2, and increases cell proliferation and angiogenesis [2]. Importantly, integrin αvβ3has been implicated in prostate cancer metastasis [14].

The inverse association between TSH and risk appeared restricted to older men and was stronger among men who smoked less, although the interaction with smoking was not statistically significant. However, the observed smoking interaction is biologically plausible given that smoking has been shown to be associated with higher T4 levels as well as lower TSH and a lower risk of hypothyroidism [15]–[18]. Recent studies have shown that smoking is associated with lower levels of thyroid autoantibodies, suggesting that smoking may be inversely associated with the autoimmune process that is hypothesized to cause thyroid dysfunction [16]. We also observed that the positive association between T4 and risk of prostate cancer was stronger among men supplemented with β-carotene, a finding that may have a biological basis. Retinol binding protein 4 (RBP4) transports retinol (which is formed from the centric cleavage of β-carotene) in circulation and complexes with transthyretin (TTR), one of the carrier molecules of T4 [19]. Given that serum retinol was 6% higher in the men supplemented with β-carotene [20], upregulation of both RBP4, and as a result, its dimer complex with TTR, is possible. Although this is speculative and the T4-prostate cancer-β-carotene interaction we found may be due to chance, further examination of the biological interrelationship between β-carotene and thyroid hormones would be useful.

Strengths of our study include our measurement of thyroid hormone concentrations in one CLIA-certified clinical laboratory using prospectively-collected serum, good quality control findings, exclusion of cases diagnosed within 3 years of blood collection to minimize reverse causation, and our detailed information regarding potential confounding factors. Our investigation was also limited in some respects. The study population was limited to smokers based on the original hypotheses and design of the parent ATBC prevention trial. There is evidence, however, that smoking lowers serum TSH and may increase the risk of a hyperthyroid state while decreasing the risk of hypothyroid disease [15]–[18]. Although we observed no interaction (*p = 0.47*) between smoking intensity (i.e., cigarettes smoked daily) and T4, we found that the inverse association between TSH and prostate cancer was stronger among men who smoked less. If true, this finding suggests that our results may have been attenuated by the smoking status of our population, and that a stronger hypothyroid-prostate cancer relation may exist in non-smokers. It should also be noted, however, that our findings are consistent with those from the one previous study that included both smokers and non-smokers [10]. Although we examined aggressive prostate cancer cases separately, we had limited statistical power to detect modest associations with aggressive disease, and were unable to examine the association between hypo- or hyperthyroid status and risk of aggressive disease, something that larger studies should address. We were only able to examine thyroid hormone levels from blood collected at one time point, which may or may not be representative of an individual's usual hormone levels throughout adulthood or hormone levels during the etiologically relevant time period.

In this prospective investigation of smokers, a hypothyroid hormonal state was associated with a lower risk of prostate cancer. Future studies should be conducted in other populations, particularly non-smokers and other ethnic-racial groups, and should specifically evaluate the association between clinical and subclinical hypothyroid status and aggressive prostate cancer.
